# A Compact Dual-Polarized Vivaldi Antenna with High Gain for Tree Radar Applications

**DOI:** 10.3390/s24134170

**Published:** 2024-06-27

**Authors:** Kaixuan Cheng, Yee Hui Lee, Jiwei Qian, Daryl Lee, Mohamed Lokman Mohd Yusof, Abdulkadir C. Yucel

**Affiliations:** 1 School of Electrical and Electronic Engineering, Nanyang Technological University, 50 Nanyang Ave., Singapore 639798, Singapore; chen1519@e.ntu.edu.sg (K.C.); qian0069@e.ntu.edu.sg (J.Q.); 2 Centre for Urban Greenery and Ecology, National Parks Board, 1 Cluny Rd., Singapore 259569, Singapore; daryl_lee@nparks.gov.sg (D.L.); mohamed_lokman_mohd_yusof@nparks.gov.sg (M.L.M.Y.)

**Keywords:** dual-polarization antenna, ground-penetrating radar (GPR), high gain, tree radar, ultra-wide bandwidth, Vivaldi antenna

## Abstract

A dual-polarized compact Vivaldi antenna with high gain performance is proposed for tree radar applications. The proposed design introduces an array configuration consisting of two pairs of two Vivaldi elements to optimize the operating bandwidth and gain while providing dual-polarization capability. To enhance the gain of the proposed antenna over a certain frequency range of interest, directors and edge slots are incorporated into each Vivaldi element. To further enhance the overall antenna gain, a metal back reflector is used. The measurement results of the fabricated antenna show that the proposed antenna achieves a high gain of 5.5 to 14.8 dBi over broadband from 0.5 GHz to 3 GHz. Moreover, it achieves cross-polarization discrimination larger than 20 dB, ensuring high polarization purity. The fabricated antenna is used to detect and image the defects inside tree trunks. The results show that the proposed antenna yields a better-migrated image with a clear defect region compared to that obtained by a commercial Horn antenna.

## 1. Introduction

Ground-penetrating radar (GPR) is the preferred technology for quick detection and imaging of tree defects, therefore dubbed as tree radar, since it does not require the installation of nails or penetration of drill bits during measurements [1] on a circular trajectory [2,3] or on a straight trajectory [4,5] away from the tree trunk. To ensure enough penetration into high water-content tree trunks, reduce labor-intensity during measurements, and achieve high-quality images of tree interiors, four aspects are to be considered for tree radar antenna design: a wide frequency band, a narrow beam with high gain, dual-polarization, and portability. Based on the literature review [5] and past experiences [1,2,3], tree radar antenna systems should be designed to operate over a wide frequency band, specifically ranging from 0.5 to 3 GHz [1,2,3], ensuring high-resolution imaging capability and deep penetration. A narrow beamwidth should reduce reflections from nearby obstacles since the target is relatively small, and high gain performance is critical to ensure maximum energy directed toward the tree trunk [6]. Compared with conventional tree radar applications, which use single polarization [2,3], polarimetric data obtained by utilizing a dual-polarized antenna is shown to be more effective in detecting trunk defects [7]. Finally, a compact antenna size is crucial for tree radars for ease of operation on-field since the operators often need to carry these antennas to perform their measurements.

However, to the best of our knowledge, there is no antenna system specifically designed for tree radar applications. Existed studies for detecting and imaging tree defects inside tree trunks have used conventional GPR antennas, which could not satisfy all specifications. To be specific, bow-tie antennas are lightweight, however, they have wide beam angles with an omnidirectional radiation pattern and a relatively low gain performance [8]. Planar antennas are portable yet may not achieve the desired high gain in the desired frequency band for tree radar applications [9]. Horn antennas [10] can provide a dual-polarized system with high gain. Nevertheless, they have large apertures, and the all-metal configuration makes them heavy and not easily portable.

Vivaldi antennas [11,12,13] can be ideal candidates to achieve a wide operating bandwidth for tree radar applications. However, the state-of-the-art Vivaldi antennas cannot satisfy all the abovementioned specifications simultaneously, which lays out critical issues remaining unsolved.

1. Many approaches have been proposed to achieve gain enhancement for Vivaldi antennas, but they may cause a sacrifice in the antenna size and the operating frequency range. Zero-index metamaterial arrays are introduced to adjust the refraction index to mitigate reflections [14,15], but they are only effective over a limited bandwidth. Besides, the size of the metamaterial depends on the operating frequency and becomes excessively large at low frequencies. Although dielectric lenses [16] are added to correct the phase errors, they come with a sacrifice of a large antenna size. While double-slot Vivaldi antennas and elliptical slot edges are proposed in [14,16], these techniques often increase the antenna size and/or decrease the operating bandwidth.
2. Existing dual-polarized Vivaldi antennas have limited gain performance and port isolation. They are commonly realized via a cross-shape configuration, where two elements are orthogonally inserted into each other [11,17,18]. The port isolation and gain performance of these antennas are around 20 dB and 5.5 dBi, respectively, which still indicates room for improvement. Besides, such a configuration requires careful design of the feeding structure since they interact with each other.
3. Antenna miniaturization for Vivaldi antennas usually comes with gain reduction and a sacrifice in radiation efficiency. To reduce the antenna size, exponential strip arms [19] are implemented to extend the low-end operating frequency without increasing the size. The exponential strip arms act like dipole antennas, introducing more frequency resonances and widening the frequency range. However, this enhancement causes a decrease in the gain performance. Loading resistance is another option [20], but the resistor reduces the radiation efficiency. A coplanar waveguide with a specifically designed feeding structure [21] helps in miniaturization, but the size of the antenna remains around half of the wavelength at the lowest frequency, limiting the portability of the radar system in practical applications.

To overcome the abovementioned issues in tree radar applications, this study proposes a compact and dual-polarized Vivaldi antenna system with a small aperture, high gain, and broadband operation, specifically working for tree radar applications [22]. Three unique design principles are introduced in this study: (1) The proposed antenna has a shared-aperture configuration to achieve dual polarization with wide bandwidth while maintaining a compact size and enhancing gain performance. (2) Directors and slots are carefully designed to improve the gain performance of antenna elements without sacrificing the broad bandwidth. (3) To further enhance the gain at low frequencies while suppressing the back lobe, a metal reflector is introduced. The proposed antenna system has been fabricated, and its performance metrics have been measured. It is shown that the proposed antenna with a compact size of 0.29 × 0.29 × 0.48 λ^3^ operates over the frequency range of 0.5 to 3 GHz, where λ is the wavelength at the lowest frequency in the band. The proposed antenna has a gain of 5.5 to 14.8 dBi from the lowest to the highest frequency in the band while achieving high cross-polarization discrimination (XPD) over 20 dB. Further validation of the fabricated antenna is performed by successfully detecting and imaging a tree defect inside a real tree trunk. The tree interior images obtained via the proposed antenna have clear defect regions with an 11 dB improvement of signal-to-clutter and noise ratio (SCNR) in the processed B-scan and a 24.4% improvement in the root mean square (RMS) of the migrated image, compared to those obtained by a commercial Horn antenna. To the best of our knowledge, the proposed antenna is the first to satisfy all the requirements of a tree radar (i.e., the frequency band of operation, high gain, dual polarization, and portability). The tree radar realized by the proposed antenna can operate on a straight or circular trajectory for tree defect detection and imaging.

The rest of this paper is organized as follows. Section 2 provides the design principles and details of the proposed antenna. The performances of the designed and fabricated antenna are provided and compared with the existing antennas in Section 3. The application of the proposed antenna to the tree interior imaging is expounded in Section 4. Finally, the conclusions are drawn in Section 5.

## 2. Antenna Design Principles

**Figure 1:**
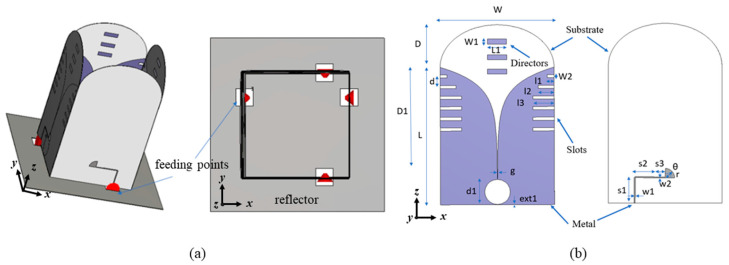
Configuration of the proposed antenna system: (**a**) dual-polarized antenna system and (**b**) single element.

The proposed dual-polarized antenna system consists of four elements with a shared aperture, as shown in Figure 1a. Every single polarization is realized by a two-element array configuration, where two elements are positioned parallel. Two sets of this single-polarization antenna are then placed orthogonally to form a dual-polarized antenna system. A metal reflector is placed at the bottom of the antenna system.

Details of the single Vivaldi element are illustrated in Figure 1b. The single element is placed on a substrate with a relative permittivity of 4.4, a loss tangent of 0.0025, and a thickness of 1 mm. To enhance the antenna’s directivity, three directors and five slots are added to the antenna element, as shown in Figure 1b. The microstrip-to-slot line with a radial stub [18] on the bottom layer is used as the antenna feed while maintaining an ultra-wide bandwidth matching. The parameters related to the design are provided in Table 1.

The major performance enhancement of the proposed design is achieved by using a shared-aperture configuration, introducing the directors and slots, and utilizing a metal reflector, as explained below.

### 2.1. The Shared-Aperture Configuration

**Figure 2:**
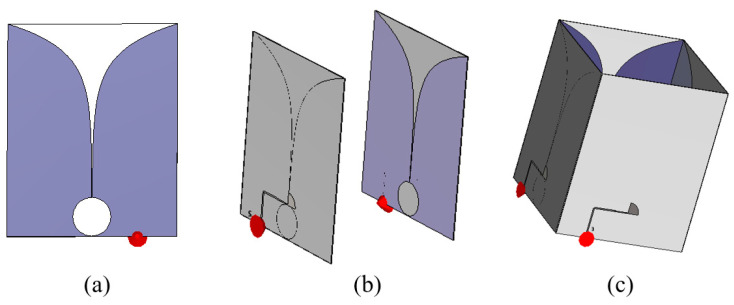
Configuration of (**a**) single Vivaldi element, (**b**) single-polarization antenna, and (**c**) shared-aperture dual-polarized antenna system.

The shared-aperture configuration can widen the operating frequency bandwidth and increase the antenna system’s gain performance. In the proposed design, a single Vivaldi antenna element [Figure 2a] is positioned parallel to another Vivaldi element with a spacing of one element’s width [Figure 2b]. These two elements in an array configuration are excited together to be operated for a single polarization. The feeding structures are in the same direction to ensure that the two ports receive identical phase excitation. The symmetry of the two elements prevents distortion in the radiation pattern. Next, two sets of the single-polarization antenna are positioned orthogonally to each other, forming a dual-polarization antenna system [Figure 2c].

**Figure 3:**
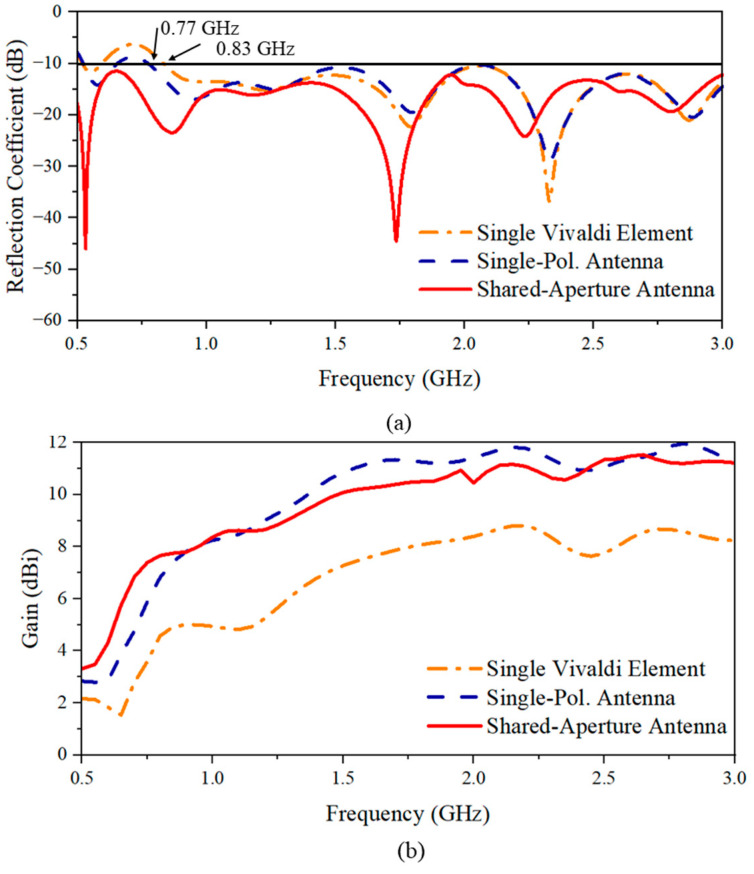
Performance comparison of single Vivaldi element, single-polarization antenna, and shared-aperture antenna: (**a**) reflection coefficient and (**b**) gain.

For the single-polarization antenna, the mutual coupling between the two antennas introduces mutual radiation resistance, which is the real component of the mutual impedance in the proposed design [23]. This mutual radiation resistance could, when viewed from the input end of the antenna, increase the equivalent real part of the impedance and reduce the imaginary component, thereby expanding the antenna’s operation bandwidth. The reflection coefficient shown in Figure 3a shows an extension of the minimum operating frequency from 0.83 to 0.77 GHz for the single-polarization configuration compared to the single element. Furthermore, the single-polarization antenna configuration also enhances the overall gain compared to a single element. This is due to the array configuration that enhances the gain in the forward direction. From the gain comparison results [Figure 3b], the gain of the single-polarization antenna shows an increase in gain by around 2 to 4 dBi compared with that of the single Vivaldi element over the entire bandwidth.

**Figure 4:**
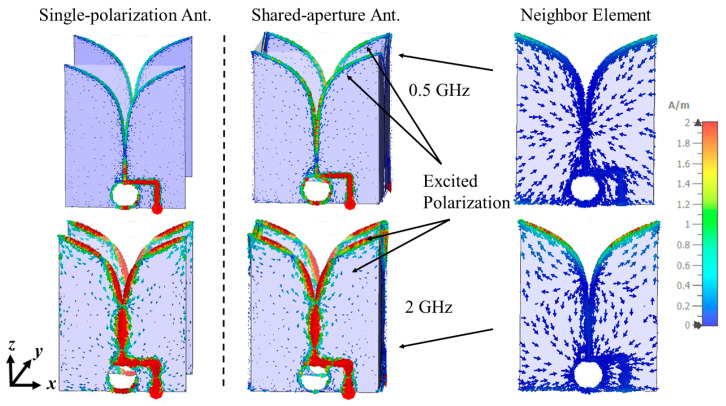
Surface currents on the single-polarization antenna and shared-aperture antenna system at 0.5 GHz and 2 GHz.

**Figure 5:**
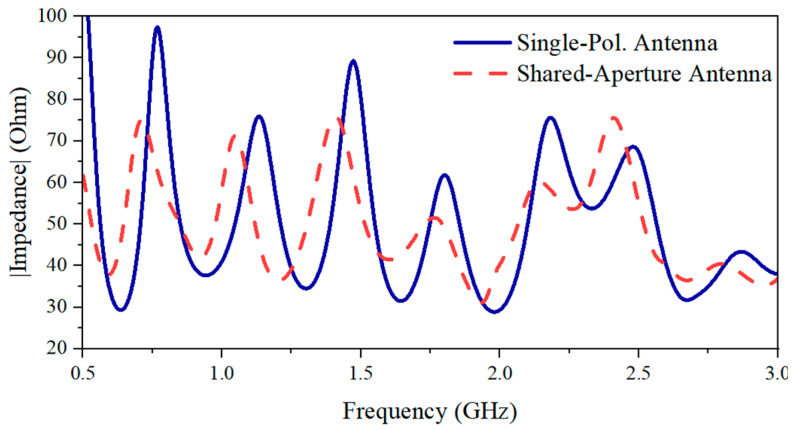
Impedance comparison between the single-polarization antenna and the same polarization in the shared-aperture configuration.

To form the dual-polarized antenna system, two sets of the single-polarization antenna are placed orthogonally in a shared-aperture configuration. The polarization of a single Vivaldi antenna element is linear, and the E-plane is parallel with the radiating tapered slots (*xoz*-plane, as indicated in Figure 1b). By constructing the shared-aperture configuration, both the horizontal (*xoz*-plane, as indicated in Figure 1a) and the vertical polarizations (*yoz*-plane, as indicated in Figure 1a) are achieved, allowing for a dual-polarization system for tree radar applications. When performing the simulations and measurements, the horizontal polarization is excited by Port 1, while Port 2 is used to excite the vertical polarization. From Figure 3a, it can be seen that the shared-aperture dual-polarization configuration further reduces the lowest operating frequency to the desired lowest frequency of 0.5 GHz. However, the gain performance is decreased by 1.1 dB within the frequency range from 0.9 to 3 GHz. By analyzing the current distribution at 2 GHz [Figure 4], it is observed that the currents leak to the neighbor elements of orthogonal polarization, which can help slightly reduce the operating frequency bandwidth due to the extension of the current length. However, it causes less concentration of the currents on the excited polarization, resulting in a gain reduction. This phenomenon can also be seen at 0.5 GHz in Figure 4. Nevertheless, better impedance matching (shown in Figure 5) at low frequencies results in enhanced radiation efficiency of the antenna system.

### 2.2. The Design of Directors

Modifications on a single Vivaldi element are performed to further increase the gain performance and compensate for the gain reduction around the center frequency. To this end, metal planar directors parallel to the orientation of the electric field are introduced at the center and in front of the radiating slots. Such slots allow for concentrating and guiding the current in the end-fire direction [24], while the currents in a Vivaldi element without slots are usually distributed along the edge of the tapered slot, the edge of the antenna, and the patch areas [25]. Additionally, the set of directors functions as an *RLC* resonator [13]. By determining the electrical size of the directors properly, the return loss of the antenna element could be further reduced, resulting in a higher gain. To achieve such concentration, guidance, and resonance, the planar directors’ area, positions, and length should be carefully determined since those also affect the input impedance of the antenna.

**Figure 6:**
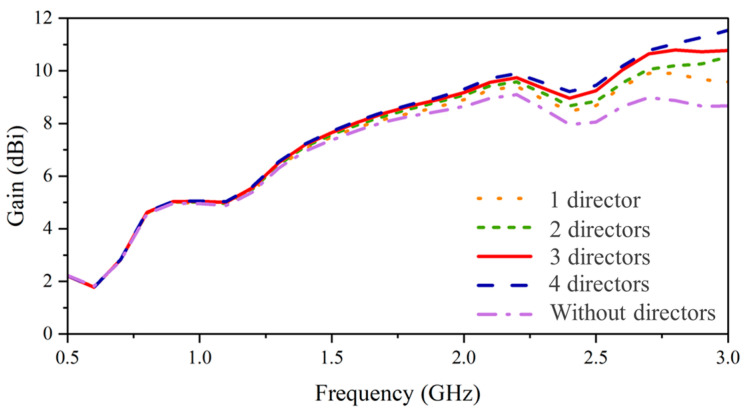
Comparison of gain for different numbers of directors.

To enhance the gain and directivity at the center frequency, the length of the directors is chosen according to the wavelength (λ_c_) of 0.1714 m. Initially, the length of the director is set to λ_c_/6 while varying the number of directors. The gain performance with respect to the number of directors is shown in Figure 6. The gain increases noticeably after 1.5 GHz, and the enhancement increases with the number of directors, especially at a frequency range above 2.5 GHz. As for the gain performance around the target frequency point, three and four directors give similar enhancements. To this end, the configuration of three directors is chosen.

**Figure 7:**
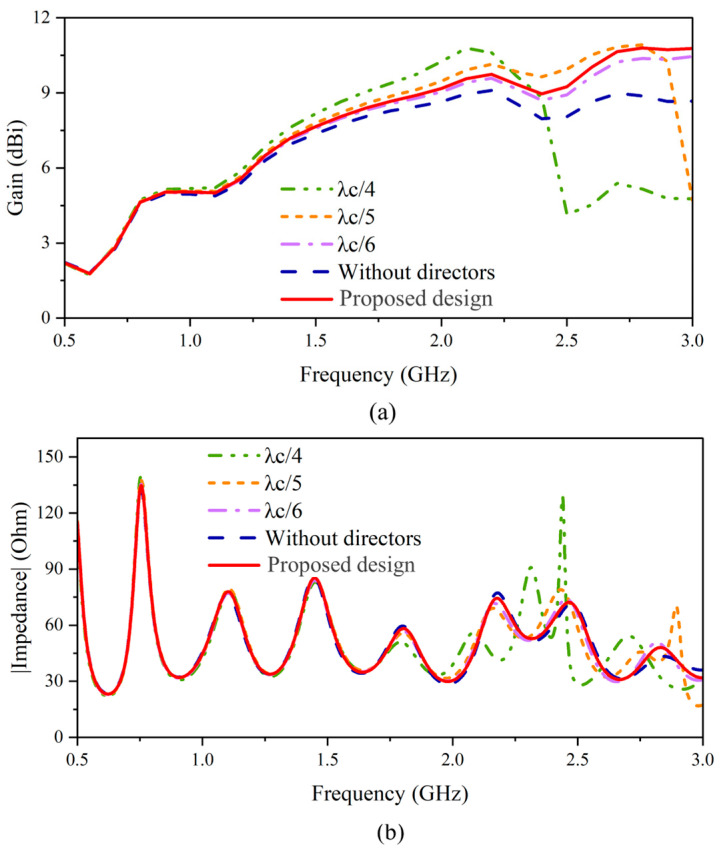
Comparison of (**a**) gain and (**b**) impedance for different lengths of directors.

To specify the optimal length of the directors, three different lengths of λ_c_/4, λ_c_/5, and λ_c_/6 are chosen. Figure 7a demonstrates the gain enhancement with respect to the length of directors. When the length reaches λ_c_/4, the parallel placed directors excite resonance at the center frequency and, therefore, enhance the radiation around the center frequency significantly. However, for the frequency range after 2 GHz, the phase-advancement of EM waves generated by such a large electrical length deteriorates the radiation characteristics, induces the impedance mismatch (130 Ω around 2.4 GHz), and degrades the gain performance [Figure 7]. Similarly, such a phenomenon is observed at 2.8 GHz when the length is λ_c_/5. Therefore, the length ranging from λ_c_/6 or longer but shorter than λ_c_/5 merits consideration. After weighing gain enhancement, the length has been finalized to around 0.175 λ_c_, which enhances the gain across the entire frequency band while maintaining impedance matching.

**Figure 8:**
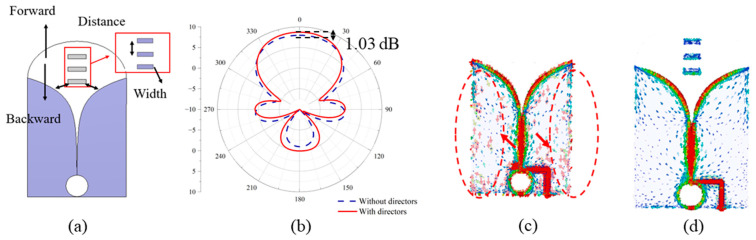
(**a**) The parameters of the directors to be optimized, (**b**) the radiation patterns with and without directors at 1.75 GHz, (**c**) the surface currents without directors, and (**d**) the surface currents with directors induced at the center frequency (1.75 GHz).

The parameters of the directors that affect the impedance and gain of the antenna include the distance between directors and the position of the director array in the single-antenna element [Figure 8a]. When the distance between directors is small, the proximity of the two directors introduces capacitive coupling, resulting in a mismatch for the antenna elements. As for a large distance, the size of the antenna is not compact. When it comes to the position, if the directors are too close to the tapered slots (moving in the backward direction, as shown in Figure 8a), the small distance between the directors and the tapered slot affects the impedance. On the other hand, if the directors are far away from the slots (moving in the forward direction, as shown in Figure 8a), they no longer serve as “directors”. It is worth noticing that the width of the directors does not significantly affect the impedance and gain performance. To keep the antenna compact, the width and spacing among directors are set to be 8 mm and 15 mm, respectively. This selection ensures gain enhancement and impedance matching. The comparison between radiation patterns with and without directors [Figure 8b] indicates that the gain enhancement by adding directors achieves 1.03 dB. The analysis of surface currents [Figure 8c,d] also indicates that the directors yield effectively concentrating and guiding the currents in the end-fire direction. The strong currents at the edge of the antenna element (within the red circles in Figure 8c) are substantially reduced with the introduction of directors.

### 2.3. The Design of Slots

The gain at low frequencies is much lower than that at high frequencies. Since the low-frequency components can deeply penetrate the tree trunks, enhancing the gain performance at low frequencies is of paramount importance. Therefore, edge slots are introduced to improve the radiation characteristics in two aspects: inducing currents along edge slots [26] and eliminating edge currents and concentrating currents along tapered slots.

The widths, spacings, numbers, and lengths of slots are to be carefully considered to enhance the gain performance. The width of the slots does not affect the radiation performance much as long as it is not too small. Once it is as small as 2 mm, the slots do not yield gain enhancement. The distance between slots determines their total area in the radiating metal board. If the distance is less than 5 mm, the slots are close to each other and require cutting a large portion of the metal, which degrades the gain performance. After optimization, the width, spacing, and number of slots are set to 5 mm, 12 mm, and 6, respectively, to balance the gain performance and impedance matching.

**Figure 9:**
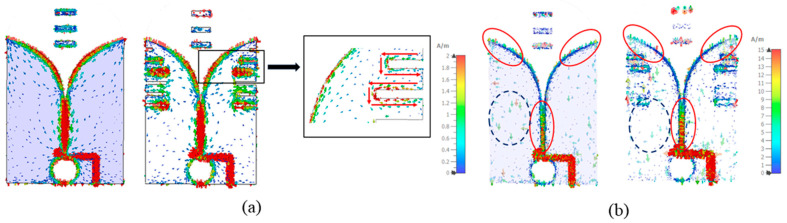
(**a**) Demonstration of currents along the edge slots at 1.75 GHz and (**b**) current flow along the tapered slots (within red solid-line circles) and edge areas (within the circle with a dashed blue line) before and after the implementation of directors at 1.75 GHz.

**Figure 10:**
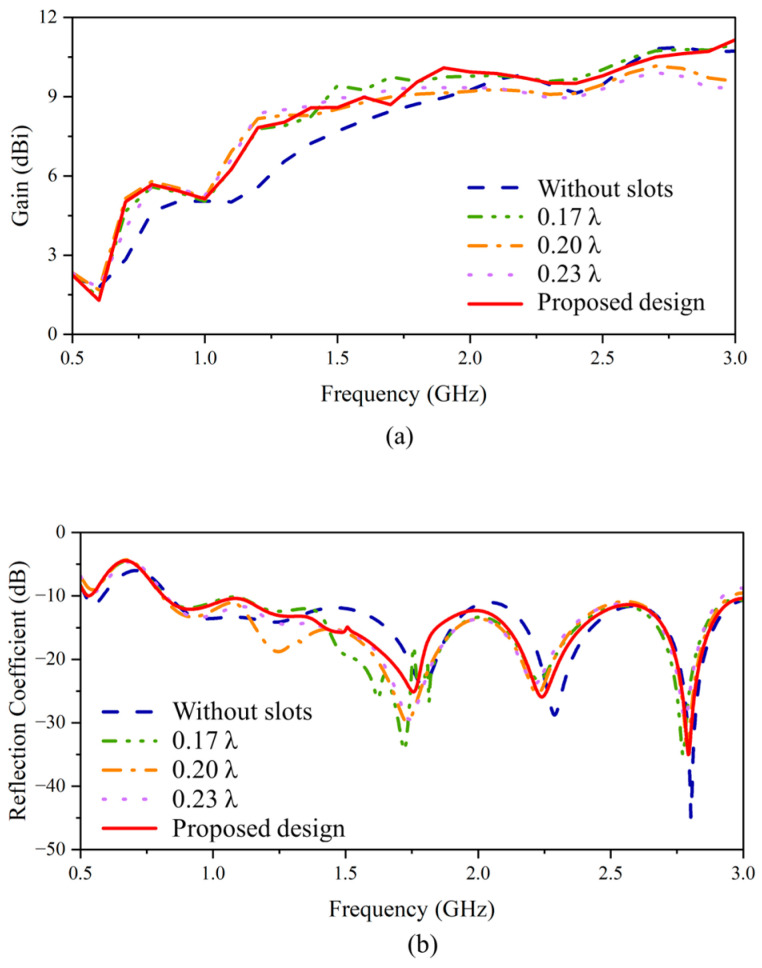
The (**a**) gain performance and (**b**) reflection coefficient after the slots with different lengths are introduced.

By introducing the slots, additional currents are induced along the slots, as shown in Figure 9a. Such currents contribute to a more directive radiation pattern [26]. However, the length of the slots should be designed carefully. These slots could lengthen the current path, which changes the phase. For the operating frequency, it should be noticed that if the length is over half of the wavelength, it could cause a phase-advancement effect. To avoid this issue, the length should be set to less than a quarter of the smallest wavelength within the target frequency range from 0.5 GHz to the center frequency of 1.75 GHz. Based on this consideration, the length of these slots is determined from a range of lengths less than 0.25 λ at 1.75 GHz, specifically 0.17, 0.2, and 0.23 λ. The evaluation of gain increments, as depicted in Figure 10a, reveals that the longer the length, the better the gain improvement at low frequencies (below 1.5 GHz). Between 1.5 GHz and 2 GHz range, the shorter length of 0.17 λ provides the best performance. As for the frequency above 2 GHz, the long slot length cannot yield a gain enhancement and can cause a reduction in the gain of around 2–3 dB. This distortion can also be seen in the S-parameters [Figure 10b]. This is because the upper two slots almost reach the tapered slots, which affects the impedance for the slot lengths of 0.2 and 0.23 λ.

To keep the return loss with S-parameters lower than −10 dB with a gain enhancement in the low-frequency range, the upper two slots are optimized to a shorter length while the remaining four slots have a length of 0.2 λ. This configuration can keep the gain enhancement from the long length and prevent the slots from being close to the tapered slots. The first and second slots are determined to be 0.15 λ and 0.07 λ long for optimizing the gain and the reflection coefficient. It is clear from the results in Figure 10 that the final slot configuration can yield a gain improvement even before 2 GHz while maintaining an unchanged gain in the high frequencies and S-parameters lower than −10 dB.

Additionally, the cut slots allow not only concentrating the current flow along the exponentially tapered slots partially but also reducing the surface currents on the patch areas around the edge to some extent [Figure 9b], resulting in an improvement in the gain performance of the proposed design.

### 2.4. The Metal Reflector

**Figure 11:**
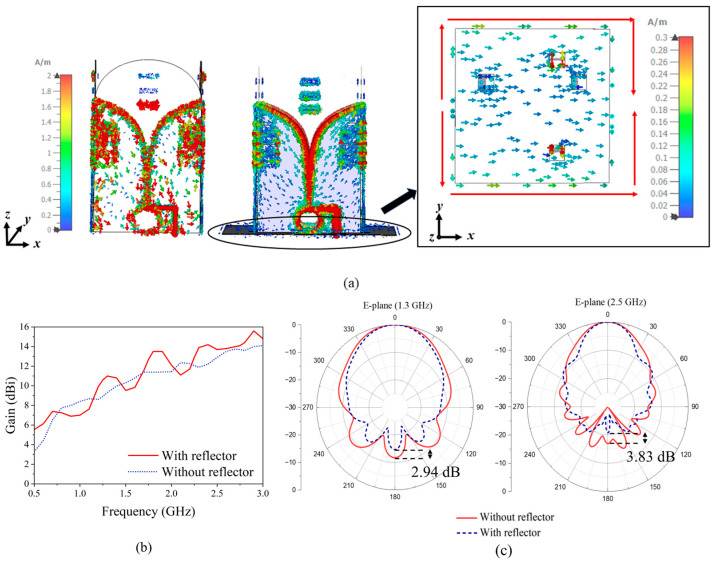
Comparison of the results with and without reflector: (**a**) The surface currents at 0.5 GHz, (**b**) gain, and (**c**) normalized radiation patterns.

Metal reflectors are often introduced to improve the gain and reduce the back lobe. In the proposed design, a reflector is placed at the back of the antenna system. The width of the reflector is 0.5 λ at 0.5 GHz, and its thickness is 1 mm. The reflector is closely attached to the end of the radiating elements, which has four small square holes (with 1.5 cm edge length) to allow cables to pass through. SMA connectors are modeled to ensure the accuracy of the simulation. Figure 11a illustrates the impact of the reflector in terms of current paths. Newly formed currents can be seen on the reflector, while the strong currents have a path length of approximately 1 λ at 0.5 GHz. The radiation from the currents on the metal reflector and the currents on the elements all together improves the gain performance. With the reflector, currents are concentrated more on the tapered slots, with fewer currents flowing back. The gain effect caused by the reflector is plotted in Figure 11b. It is shown that the reflector can increase the gain at most of the frequency points, especially at the lowest frequency points, around 0.6 GHz. The width of the reflector is chosen according to the lowest operating frequency. Therefore, the phase of the currents on the reflector varies with the frequency, leading to gain fluctuations within the operating band. As shown in Figure 11b, the gain decreases negligibly around 1, 1.5, and 2.1 GHz. However, the degradation is within an acceptable range. Furthermore, the reflector also suppresses the back lobe. In Figure 11c, the front-to-back lobe ratios of the radiation pattern (*xoz*-plane) at 1.3 GHz and 2.5 GHz are reduced by 2.94 dB and 3.83 dB, respectively.

## 3. Antenna Performance

**Figure 12:**
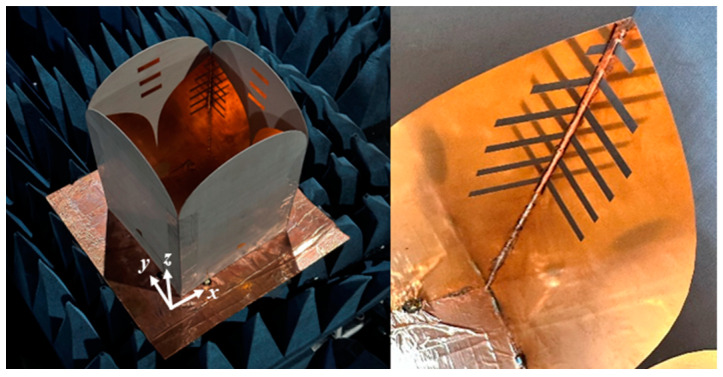
The fabricated shared-aperture antenna system.

**Figure 13:**
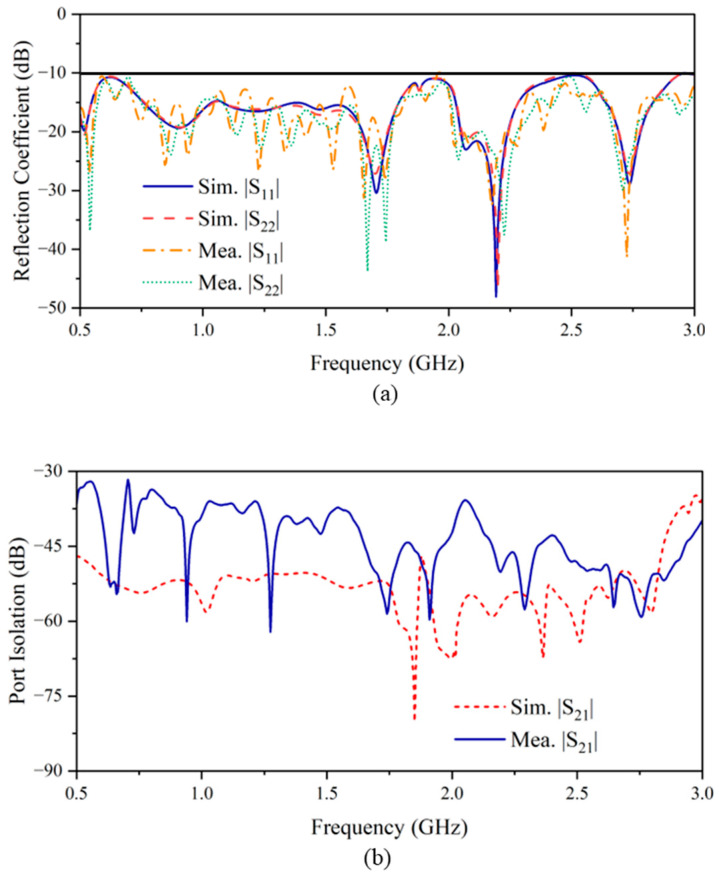
The simulated and measured (**a**) reflection coefficient and (**b**) port isolation.

The fabricated antenna [Figure 12] consists of two pairs of Vivaldi elements perpendicular to each other and is backed by the reflector, realized by a copper foil-covered cardboard. Four small squares are cut off from the reflector to allow cables go through and feed the radiating elements. A power divider is used to feed the parallel elements with the same phase simultaneously. To measure the reflection coefficient and the port isolation, the antenna is placed in the anechoic chamber facing directly toward absorbers to avoid environmental inference. The simulated and measured reflection coefficients of each polarization are shown in Figure 13a. It is observed that the antenna has satisfactory performance. From 0.5 to 3 GHz, the reflection coefficients are lower than −10 dB. The results from the measurement match those of the simulation. The port isolation exceeds 30 dB across the entire bandwidth [Figure 13b], while the discrepancies between the simulation and measurement results can be attributed to the imperfections in the soldering process and utilization of a power divider.

**Figure 14:**
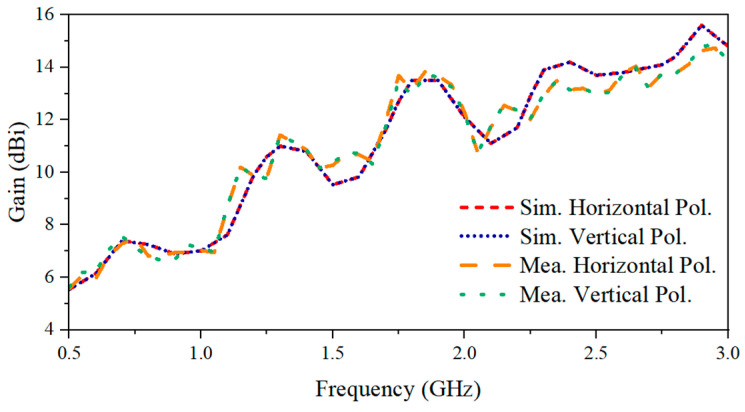
The simulated and measured gain for two polarizations.

**Figure 15:**
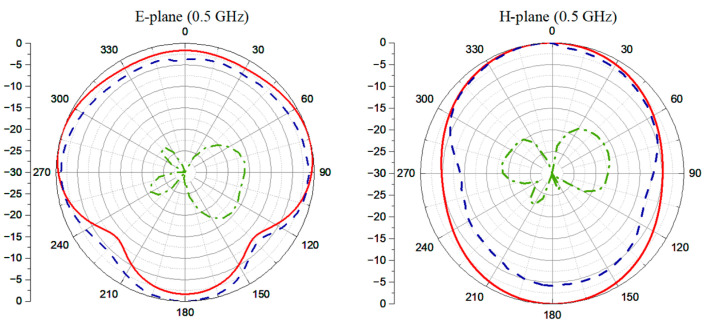

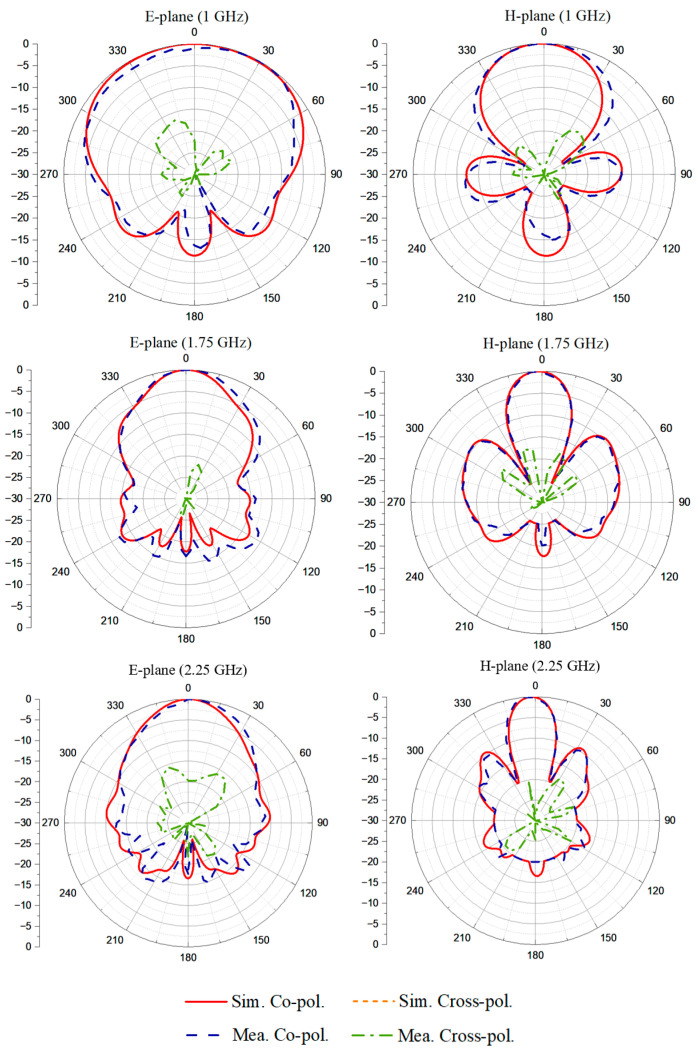
Normalized radiation pattern on E and H planes.

**Figure 16:**
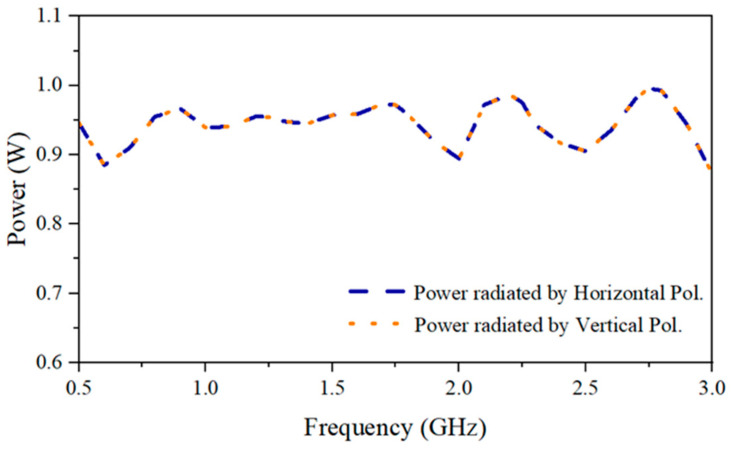
The simulated radiated power by horizontal and vertical polarizations.

Gain is measured by referencing a standard antenna. Both the standard and the proposed antennas serve as individual transmitters and are alternatively oriented toward the same receiving antenna, with two individual transmission coefficients being recorded. The difference between the two measured S-parameters represents the gain difference between the proposed antenna and the standard antenna. As a result, the gain of the tested antenna is calculated by utilizing the identified gain performance of the standard antenna. The gain characteristic over the whole bandwidth is shown in Figure 14. The gain exhibits a general increasing trend with frequency, ranging from 5.56 to 14.8 dBi for measurement results, which matches well with those of the simulation. Because of the symmetric configuration, the horizontal and vertical polarizations have identical radiation patterns on the E- and H-planes. Thus, the radiation patterns of one polarization are shared here to demonstrate the performance of the fabricated antenna system. The E-plane (*xoz*-plane) here represents the plane parallel to the excited polarization, while the H-plane (*yoz*-plane) represents the plane vertical to the polarization. To measure the radiation pattern, a standard antenna was placed in the far-field region of the proposed Vivaldi antenna system. To record the radiation pattern along various directions, the Vivaldi antenna was rotated 360° with a stepping angle of 5°. At each rotation angle, the transmission coefficient of these two antennas, represented by S_12_, was recorded, resulting in a total number of 72 sampling points. Then, the normalization of the collected data was performed to plot the radiation pattern in polar coordinates. In the measurement, the normalized horizontally polarized radiation pattern was measured at 0.5 GHz, 1 GHz, 1.75 GHz, and 2.25 GHz, as shown in Figure 15. The measurement results are similar to the simulation results, and the maximum XPD calculated via

(1) $$XPD=\frac{E_{co-pol}}{E_{cross-pol}}$$

is 20 dB at 2.25 GHz. The simulated XPD exceeds 30 dB, which is not illustrated in the plotted figures since the lowest range is set to −30 dB. As for the measurement, it is important to note that the XPD being higher than 20 dB indicates a good isolation between the two polarizations. According to [12], the radiation efficiency should be higher than 80% to ensure the antenna’s capability in real application scenarios. The simulated power radiation by horizontal and vertical polarizations of the proposed antenna system is shown in Figure 16. It can be seen that the radiation efficiencies of both polarizations are higher than 87% through the operating frequency range, indicating the satisfactory performance of the proposed antenna system.

The performance comparison among the proposed dual-polarized Vivaldi antenna and other antennas proposed in the literature is provided in Table 2. It is clear from the table that the proposed antenna has good isolation, high XPD, compact size, narrow beamwidth, and high gain performance.

Unlike conventional GPR applications for underground target detection, tree trunk radar application needs a narrow beam with high gain performance. Thus, even though the design in [11] has a wider operation bandwidth and a smaller aperture, the gain is significantly lower than that of the proposed design. The antenna in [12] has a comparable size, but the proposed antenna has a higher gain. As for the design in [27], both the gain and the size fail to reach those of our antenna. Moreover, the comparisons between the proposed antenna and [17,28] reveal that the proposed antenna system performs better in all parameters. The gain of the antenna system in [13] is 0.4 dB higher than that of the proposed one, which is achieved by an “H” shape metasurface. However, its size is too large compared to ours. Additionally, the comparison of 3 dB beamwidth is also included. Since the referenced antennas work in different frequency ranges, the results for the common frequency point (1 GHz) presented in each paper are chosen. From the comparison, it is concluded that the proposed antenna has a relatively narrow beam. To sum up, the proposed antenna system performs best in all the key characteristics (i.e., the frequency band of operation, high gain, dual-polarization, and portability) for tree defect detection and imaging compared to existing antenna systems reported in the literature.

## 4. Tree Trunk Scanning Validation

**Figure 17:**
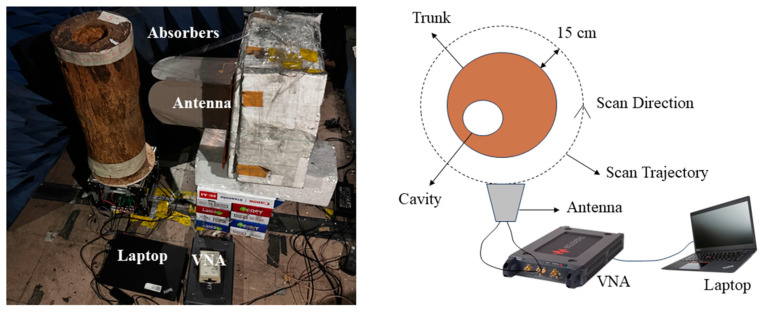
The setup for scanning the real tree trunk via the proposed antenna system.

To validate the functionality of the designed antenna system for tree defect detection and imaging, a measurement was performed using the proposed antenna on a real tree trunk. The target tree trunk, obtained from a rain tree in Singapore, has a diameter of around 30 cm and a 6 cm diameter cavity located 5 cm away from the trunk center [Figure 17]. The size of the trunk is quite similar to that of trees in urban areas. However, due to the high humidity in Singapore, the water content of these tree trunks tends to be high, and therefore, they have higher relative permittivity and conductivity than dry wood.

The dual-polarized antenna system is connected to a vector network analyzer (VNA), as shown in the measurement setup [Figure 17]. The antenna system is placed 15 cm away from the surface of the trunk to test the detection capability while operating in a contactless scanning configuration. It should be noted that the detection capability varies in terms of the distance between the antenna and the tree trunk, which is a research topic for future studies. The trunk is placed on a rotating platform and moved with a step of 9 degrees, while the antenna system is placed at a fixed position. Absorbers were placed around the tree trunk sample to suppress unwanted environmental clutter. At each position, the system records S-parameters for the vertical polarization, horizontal polarization, and cross-polarizations and transforms those to the time-domain A-scans. Forty A-scans were collected for this trunk to form a B-scan for one polarization. An ETS-Lindgren 3115 double-ridged Horn antenna was also used to collect A-scans and form B-scans for comparison purposes. The commercial Horn antenna operates from 0.75 to 18 GHz with a gain of around 3 to 9.5 dBi within the scanning band from 0.75 to 3 GHz. To ensure a rigorous comparative study, the Horn antenna is placed at the same position as the Vivaldi antenna, at the same scanning height and at the same distance between the trunk and the antenna aperture. Similarly, forty A-scans were collected to form a raw B-scan. The B-scans obtained by the proposed antenna and commercial Horn antenna were processed by signal processing techniques and a modified Kirchhoff migration algorithm. The signal-processing procedure contains the antenna calibration, a Kaiser window filter, and a C3-based time zero gating [5,29]. The antenna calibration eliminates internal reflections from the antenna system and uncovers the signatures caused by the trunk itself. A Kaiser window filter with a center frequency of 1 GHz is used to improve the signal-to-noise level. Finally, a C3-based time zero gating automatically detects and removes the reflection from air-bark surface, which is a strong clutter that hides the hyperbola signature from the cavity. With the processed B-scan, modified Kirchhoff migration can reconstruct the internal structure of the target tree trunk via [3]

(2) $$I\left(r_{m}\right)=\frac{R_{min}}{2\pi }\int_{0}^{L}\frac{\partial R}{\partial n}\left(\frac{1}{R^{2}}G\left(r,\frac{R}{v}\right)-\frac{G^{\prime }\left(r,\frac{R}{v}\right)}{vR}\right)ds$$

where *G* is the processed B-scan, *I* is the reconstructed image intensity, *r* is the projected position, *v* is the effective electromagnetic velocity, *L* is the circumference of the target trunk section, *r_m_* is the investigated point, and *R* = || *r_m_ − r*||.

**Figure 18:**
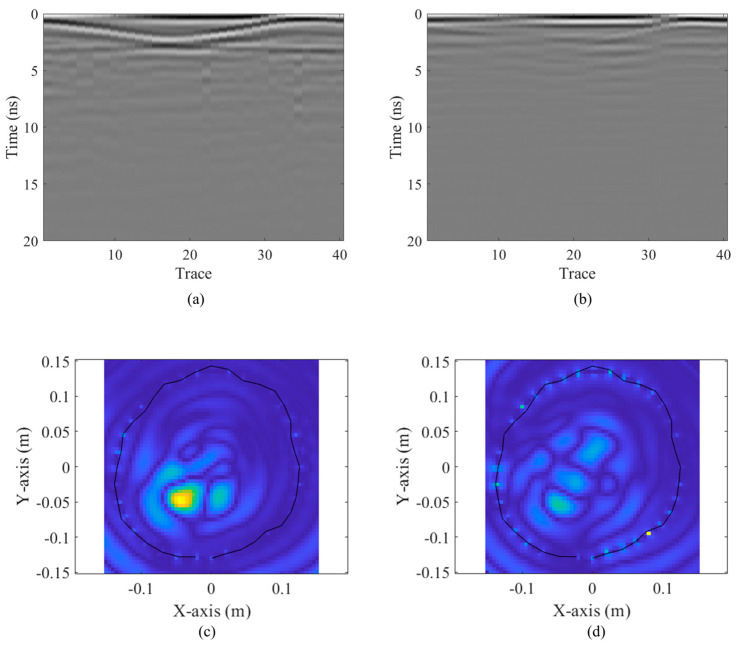
The measured B-scans obtained by (**a**) the proposed Vivaldi antenna and (**b**) the commercial Horn antenna; the migrated results obtained by (**c**) the proposed Vivaldi antenna and (**d**) the commercial Horn antenna.

The resulting B-scans and reconstructed images of tree interiors are compared in Figure 18. It is noticeable that the proposed Vivaldi antenna system yields a clearer hyperbola signature caused by the cavity in the processed B-scan and a better-reconstructed image with a distinguishable cavity region [Figure 18]. On the other hand, one cannot distinguish the cavity region easily from the processed B-scans obtained by the commercial Horn antenna. To quantify the improvement achieved by the proposed antenna system in defect detection and imaging, the SCNR [30] of the processed B-scan and the RMS of the migrated images obtained by both antennas are calculated via

(3) $$SCNR=10{log}_{10}(\frac{Signal\;Level}{Clutter\;and\;Noise\;Level})$$

(4) $$RMS=\sqrt{\frac{1}{M\times N}\sum_{i=1}^{M}\sum_{j=1}^{N}{\left(Image(i,j)\right)}^{2}}$$

where *Image* represents the matrix holding pixel values, and *M* and *N* denote the numbers of rows and columns of the matrix, respectively. It should be noted that the intensity of the imaging pixels in migrated images in these two cases are normalized to [0, 1] for a fair comparison, and the high RMS value represents a better image contrast introduced by a distinguished defect area. The results of the quantitative analysis are shown in Table 3, which indicates an over 11 dB improvement in SCNR by utilizing the proposed Vivaldi antenna. Moreover, the RMS of the migrated image obtained by the proposed antenna achieves a 24.4% enhancement, demonstrating better image clarity. All of these results verify the advantage of the proposed antenna system for tree radar applications. These results verify the advantage of the proposed antenna system for tree defect imaging. The combined co-polarization configuration [7] also shows the cavity signature, but the signal-to-noise ratio achieved by the configuration is still relatively low, which requires further study in interpreting the polarimetric data.

## 5. Conclusions

A compact dual-polarized antenna system with high gain performance was proposed in this study for tree trunk radar application. In this design, three planar directors and six edge slots were introduced to improve the gain performance of the single Vivaldi antenna. Two elements parallel to each other formed one single polarization. Two pairs of them were positioned orthogonal to each other with a shared aperture to realize the dual-polarized antenna system. A metal reflector was attached to the end of the elements to improve the gain performance further and suppress the back lobe. Analysis of the measurement results revealed that the designed antenna system with a compact size of 0.29 × 0.29 × 0.48 λ^3^ can work from 0.5 GHz to 3 GHz. The minimum XPD is 20 dB, and the measured gain varies from 5.5 to 14.8 dBi. The reconstructed tree interior images obtained by the proposed antenna and a commercial Horn antenna showed that the proposed antenna yields clear migrated images compared to existing antennas for tree trunk defect imaging and detection.

## Figures and Tables

**Table 1:** Design parameters of a Vivaldi element (unit: mm).

Symbol	Parameter	Symbol	Parameter	Symbol	Parameter
W	178.5	g	0.1	r	15
D	68	d1	40	θ	88
D1	178.5	ext1	0.1	l1	11
L	220	s1	40.5	l2	25
W1	8	s2	29.2	l3	33.55
L1	30	s3	17.75	w2	1.2
d	12	w1	1.8	W2	5

**Table 2:** Comparison of antenna performance.

Ref.	Freq. (GHz)/fBW	Isolation (dB)	XPD (dB)	Gain (dBi)	3 dB Beamwidth at 1 GHz (°)	Size (λ^3^)
[11]	0.56–7.7/173%	28	17.2	1.2–9.2	90	0.24 × 0.24 × 0.35
[12]	0.4–4/164%	40	23	4–12	NG	0.28 × 0.28 × 0.2
[27]	1.85–18.3/163%	22	16.5	4–11.3	NA	0.36 × 0.36 × 0.9
[17]	3.1–10.6/109%	20	15	Max. 10	NA	0.36 × 0.36 × 0.55
[28]	0.5–3/143%	15	NG	1–8.6	>120	0.33 × 0.33 × 0.3
[13]	0.9–4/126%	NG	NG	6.7–15.2	100	0.72 × 0.72 × 0.86
**Ant.**	**0.5–3/143%**	**30**	**20**	**5.5–14.8**	**90**	**0.29 × 0.29 × 0.48**

**Table 3:** Quantitative comparison of results obtained by the proposed antenna and the commercial Horn antenna.

	B-Scan	Migrated Image
	SCNR (dB) (⬆)	RMS (⬆)
Proposed Vivaldi Antenna	6.34	0.1371
Commercial Horn Antenna	−5.19	0.1102

## Data Availability

Data are contained within the article.
